# Assembled and annotated 26.5 Gbp coast redwood genome: a resource for estimating evolutionary adaptive potential and investigating hexaploid origin

**DOI:** 10.1093/g3journal/jkab380

**Published:** 2021-12-14

**Authors:** David B Neale, Aleksey V Zimin, Sumaira Zaman, Alison D Scott, Bikash Shrestha, Rachael E Workman, Daniela Puiu, Brian J Allen, Zane J Moore, Manoj K Sekhwal, Amanda R De La Torre, Patrick E McGuire, Emily Burns, Winston Timp, Jill L Wegrzyn, Steven L Salzberg

**Affiliations:** 1 Department of Plant Sciences, University of California, Davis, Davis, CA 95616, USA; 2 Department of Biomedical Engineering, Johns Hopkins University, Baltimore, MD 21218, USA; 3 Center for Computational Biology, Johns Hopkins University, Baltimore, MD 21211, USA; 4 Department of Ecology and Evolutionary Biology, University of Connecticut, Storrs, CT 06269, USA; 5 Department of Computer Science & Engineering, University of Connecticut, Storrs, CT 06269, USA; 6 Department of Molecular Biology and Genetics, Johns Hopkins University, Baltimore, MD 21205, USA; 7 School of Forestry, Northern Arizona University, Flagstaff, AZ 86011, USA; 8 Save the Redwoods League, San Francisco, CA 94104, USA; 9 Institute for Systems Genomics, University of Connecticut, Storrs, CT 06269, USA; 10 Department of Computer Science, Johns Hopkins University, Baltimore, MD 21218, USA; 11 Department of Biostatistics, Johns Hopkins University, Baltimore, MD 21205, USA

**Keywords:** genome assembly and annotation, coast redwood, *Sequoia sempervirens*, conifer, gymnosperm, hexaploid genome

## Abstract

Sequencing, assembly, and annotation of the 26.5 Gbp hexaploid genome of coast redwood (*Sequoia sempervirens*) was completed leading toward discovery of genes related to climate adaptation and investigation of the origin of the hexaploid genome. Deep-coverage short-read Illumina sequencing data from haploid tissue from a single seed were combined with long-read Oxford Nanopore Technologies sequencing data from diploid needle tissue to create an initial assembly, which was then scaffolded using proximity ligation data to produce a highly contiguous final assembly, SESE 2.1, with a scaffold N50 size of 44.9 Mbp. The assembly included several scaffolds that span entire chromosome arms, confirmed by the presence of telomere and centromere sequences on the ends of the scaffolds. The structural annotation produced 118,906 genes with 113 containing introns that exceed 500 Kbp in length and one reaching 2 Mb. Nearly 19 Gbp of the genome represented repetitive content with the vast majority characterized as long terminal repeats, with a 2.9:1 ratio of Copia to Gypsy elements that may aid in gene expression control. Comparison of coast redwood to other conifers revealed species-specific expansions for a plethora of abiotic and biotic stress response genes, including those involved in fungal disease resistance, detoxification, and physical injury/structural remodeling and others supporting flavonoid biosynthesis. Analysis of multiple genes that exist in triplicate in coast redwood but only once in its diploid relative, giant sequoia, supports a previous hypothesis that the hexaploidy is the result of autopolyploidy rather than any hybridizations with separate but closely related conifer species.

## Introduction

Coast redwood (*Sequoia sempervirens*) is one of the most widely recognized and iconic tree species on earth (Barbour *et al.* 2001). Its natural range is found primarily along the northern California Pacific Ocean coastline (Lorimer *et al.* 2009), although coast redwood trees have been planted all over the world. Coast redwood is the tallest of all trees (the tallest discovered to date is 115.5** **m) and among the oldest (living up to ∼2200** **years) (Lanner 1999). As currently taxonomically circumscribed, coast redwood is the only extant member of its genus *Sequoia* and is considered “endangered” by the IUCN Red List (Farjon and Schmid 2013). Each of its two closest relatives, giant sequoia (*Sequioadendron giganteum*) and dawn redwood (*Metasequoia glyptostroboides*), is also the only extant member of its genus. Coast redwood has a hexaploid genome (Hirayoshi and Nakamura 1943; Stebbins 1948; Saylor and Simons 1970; Ahuja and Neale 2002) (2*n**** ***=*** ***6*x**** ***=*** ***66), whereas giant sequoia and dawn redwood are diploids (2*n**** ***=*** ***2*x**** ***=*** ***22). Coast redwood has a very large genome (originally estimated at 31.5*** ***Gbp), approximately three times that of each of its two diploid relatives (9.7*** ***Gbp for giant sequoia and 10.8*** ***Gbp for dawn redwood) [genome size estimates compiled in Ahuja and Neale (2005), converted from previously published flow cytometry measurements in pg/C where 1** **pg*** ***=*** ***980 Mbp]. The origin of polyploidy in coast redwood has been debated for many years. Stebbins (1948) proposed that it might be an autoallopolyploid, later supported by Ahuja and Neale (2002). However, in a more recent study, Scott *et al.* (2016) argued that an autopolyploid origin was more likely.

Coast redwood forests were extensively logged (nearly 95% of virgin forest) following immigration of Europeans into California (Burns *et al.* 2018). There is currently strong interest in accelerating the return of old-growth forest characteristics including a diverse forest structure with large-stature trees. Restoration and conservation efforts rely critically on the ability to measure and monitor genetic variation. Traditionally, foresters have estimated genetic variation by measuring phenotypes in some type of field test, such as a provenance test (Kuser *et al.* 1995). This approach yields high quality data about the amounts and distribution of genetic variation across a landscape; however, this approach takes many years and tests are expensive to establish, maintain, and measure. An alternative approach is to use genetic markers. However, nearly all genetic markers developed to date in coast redwood (Ahuja 2017) reveal genetic variation in noncoding regions of the genome, thus they estimate only selectively neutral variation. The ability to interrogate genomic regions under selection and estimate adaptive genetic diversity requires genetic markers from protein-coding regions of the genome. Such markers have not existed for coast redwood and can only be developed following transcriptome or whole-genome sequencing.

Genome sequencing in conifers did not begin until 10 years after the sequencing of the human genome because of economic and technical limitations. Conifer genomes are 3–10 times larger than the human genome, and the highly repetitive content of these genomes made genome assembly challenging. Beginning in the decade 2010–2020, these limitations were gradually overcome and currently nine conifer genome sequences have been reported; Norway spruce (*Picea abies*) (Nystedt *et al.* 2013), Sitka × Engelman spruce hybrid (*Picea sitchensis ×* *Picea* *engelmannii*) (Birol *et al.* 2013), loblolly pine (*Pinus taeda*) (Neale *et al.* 2014; Zimin *et al.* 2014), white spruce (*Picea glauca*) (Warren *et al.* 2015), sugar pine (*Pinus lambertiana*) (Stevens *et al.* 2016), Douglas-fir (*Pseudotsuga menziesii*) (Neale *et al.* 2017), silver fir (*Abies alba*) (Mosca *et al.* 2019), Siberian larch (*Larix sibirica*) (Kuzmin *et al.* 2019), and giant sequoia (Scott *et al.* 2020). A key feature of many conifer species is the presence of haploid megagametophytic tissue within the otherwise diploid tissue of the seeds. In many cases, this haploid tissue can be dissected from a single seed in a large-enough quantity for complete genome sequencing, thereby simplifying the assembly. All of the genome sequences reported above used megagametophyte tissue, except for white spruce. All these genome assemblies currently are highly fragmented assemblies, with the notable exception of that for giant sequoia which employed long-read sequencing and Hi-C scaffolding to obtain 11 chromosome-scale scaffolds equaling the known chromosome number (Scott *et al.* 2020). A nearly identical sequencing strategy is used here to sequence and assemble the coast redwood genome.

As a result, we report the following: the sequencing, assembly, and annotation of the very large and hexaploid coast redwood genome; the discovery of genes for estimating evolutionary adaptive potential of coast redwood in current and future environments; and investigation of the origin of coast redwood hexaploidy.

## Materials and methods

### Reference tree and biological samples

The reference tree, SESE104, was found in the Santa Cruz Mountains, California, USA. The tree is 107.2** **m tall and is estimated to be 1390** **years old. Cones were collected for seed from which to isolate megagametophytes (1*n*) for DNA isolation and Illumina sequencing. Needles (2*n*) were collected for high molecular weight DNA isolation for Oxford Nanopore Technologies (ONT) sequencing and HiRise scaffolding.

### Genome sequencing and assembly strategy

#### DNA extraction and library preparation:

Following the protocol used recently for the giant sequoia genome (Scott *et al.* 2020), we generated two types of reads: short, highly accurate Illumina reads and long, somewhat less accurate ONT reads. The Illumina data were generated on an Illumina HiSeq 4000 instrument in high-yield mode at the UC Davis Genome Center. We used standard PCR-free library preparation after extracting megagametophyte DNA from a single seed from the SESE104 tree, following the strategy used for loblolly pine (Neale *et al.* 2014) and other conifers.

Because ONT sequencers require much more DNA than can be obtained from a single seed, we used needle tissue for DNA extraction and sample preparation to produce >20x coverage from ONT long- reads. In 2017, we collected foliage from the upper canopy of the same coast redwood tree (SESE104) from which the seed originated. From this foliage, we extracted high molecular weight DNA following the protocol described in Workman *et al.* (2018). We first isolated purified genomic DNA through a nuclei extraction and lysis protocol. The mature needle tissue was homogenized in liquid nitrogen, then added to a gentle lysis buffer (after Zhang *et al.* 2012), containing spermine, spermidine, triton, and β-mercaptoethanol) and stirred at 4°C for 10 min. Cellular homogenate was filtered through five layers of Miracloth into a 50** **ml Falcon tube, then centrifuged at 4°C for 20** **min at 1900** **×** **g, which was selected based on an estimated genome size of around 28** **Gbp. Extracted nuclei were then lysed and gDNA precipitated using the Circulomics Nanobind Plant Nuclei Big DNA kit, alpha version (SKU NB-900-801-01). Then 1** **μg of purified genomic DNA was input into the Ligation sequencing kit (LSK108-LSK109, Oxford Nanopore Technologies), according to protocol, with the exception of end repair optimization (100** **μl sample, 14** **μl enzyme, 6** **μl enzyme at 20°C for 20** **min, then 65°C for 20** **min). Samples were sequenced on R9.4 minION flowcells using either the minION or GridION for 48** **h, then raw fast5 data was basecalled with Albacore version 2.13.

Additional leaf tissue from SESE104 was sent to Dovetail Genomics (Scotts Valley, CA, USA) for preparation of proximity ligation libraries using both the Chicago and Hi-C protocols, as described by Putnam *et al.* (2016). Each of these libraries captured long-range interactions within chromosomes, which in turn allows for substantial improvement in the scaffold size of resulting assemblies. Sequencing of both libraries, yielding ∼39X genome coverage for each (Table 1), was done at the UC Davis Genome Center on an Illumina HiSeq 4000 instrument.

**Table 1:** Sequence data generated for the coast redwood genome

Data type	Average read length (bp)	Number of reads	Genome coverage^*a*^	Source of tissue
Illumina paired end	2 × 150	21,588,293,516	122x	1*n* megagametophyte
Dovetail Chicago	2 × 151	6,924,430,790	39x	2*n* needle
Dovetail Hi-C	2 × 151	6,918,097:308	39x	2*n* needle
ONT	7:775	74,815,884	22x	2*n* needle

a Coverage is computed using the estimated genome size of 26.5 Gbp as described in the following text.

#### Contig assembly:

The overall assembly followed a protocol very similar to that used for the giant sequoia genome (Scott *et al.* 2020). To create the initial contig assembly, we used MaSuRCA version 3.2.8 in its hybrid mode, which combines long and short reads (Zimin *et al.* 2017). MaSuRCA is based on the idea of producing super-reads by extending Illumina reads uniquely using k-mer graphs (Zimin *et al.* 2013). All Illumina reads that extend to the same super-read are replaced by a single copy of the super-read. The super-reads are then used to correct the ONT reads. We compute the approximate alignments of the super-reads to ONT reads and then produce mini-assemblies of exactly overlapping super reads for each ONT read that are the best representation of that read’s sequence. We call these mini-assemblies mega-reads.

For assembly we used default settings with a single-pass mega-reads construction procedure (using MEGA_READS_ONE_PASS** **=** **1 in the MaSuRCA configuration file). To manage the enormous amount of data, we interrupted the MaSuRCA pipeline at the “create_mega_reads” step. Then we split super-reads into batches and proceeded by matching each batch against the entire set of the ONT reads. First, we produced all approximate alignments of the super-reads to the ONT reads using the “jf_aligner” program in MaSuRCA. The memory usage of jf_aligner is proportional to the amount of sequence in the super-reads, and batching allowed us to use multiple compute nodes in parallel, with 1** **TB of RAM each. After all alignments were done, we merged the alignment files with the “merge_coords” program and piped its output into the “longest_path” program, which constructed the mega-reads. Normally all three of these modules are run within a single executable “create_mega_reads”, but they can be separated for unusually large assembly projects. For this task, we used a special-purpose local grid with 50 1TB RAM computers and SLURM scheduler. The memory usage per node was about 0.5** **TB and the total compute time for the mega-reads steps was about 800,000 CPU hours. The need to run in batches added significant intermediate storage and overhead expense and thus it is not used by default in MaSuRCA. We saved the output of the longest_path program into a file and resumed the execution of MaSuRCA by re-running the auto-generated assemble.sh script. We note that in the current release of MaSuRCA, version 4.0.3, the mega-reads code has been optimized for memory use and running the components separately is no longer necessary even for genomes similar in size to that of the coast redwood. After postprocessing, MaSuRCA proceeded with no further manual intervention, assembling the mega-reads with a modified version of the CABOG 8 assembler included in MaSuRCA. This assembly step took about 321,160 CPU hours. This step produced assembly version 1.0 (Table 2).

**Table 2:** Results for processing of Illumina reads into super-reads and mega-reads

Derived data type	Average read length (bp)	Number of reads	Genome coverage
Super-reads	352	212,922,309	2.8x
Mega-reads	6670	71,380,616	18x

MaSuRCA reduced the data from over 21 billion Illumina reads into ∼213 million super-reads, preserving the information contained in the Illumina data. The super-reads were then used to transform ONT reads into highly accurate mega-reads.

#### HiRise scaffolding:

We delivered the 1.0 assembly to Dovetail Genomics for scaffolding with proximity ligation data. They utilized the HiRise scaffolder (Putnam *et al.* 2016) to scaffold the contigs, using linked reads from both “Chicago” and Hi-C libraries. This process first mapped all the reads from each library to the contigs, and then linked together contigs based on the number of paired reads connecting them. Contigs with larger numbers of linking reads should be closer together in the final scaffolds, and the scaffolder attempts to optimize inter-contig distances as a function of the number of linking reads. After multiple rounds of scaffolding, this process yielded assembly version 2.1.

#### Post scaffolding processing:

We examined the scaffolds produced by the HiRise scaffolder to look for telomere and centromere sequences. We identified the sequence of the telomere to be CCCT[A/C]AA by running the Tandem Repeat Finder (TRF) software on the contigs and looking for the most common repeats. We also found the following very high-copy number 148-bp repeat sequence, which we determined to be the centromeric repeat unit: AAAAAATGAAGGTTCGCGAAAGGCGATAGAAAATACGCGTACAGAATGCACAACTACAGTGCAATCCACAATCGTAGTCACCAAAGTTGATCGATGGCCCACGAAAACTCAAACACGAGCGGTTTCCTAAATGCGGCTATAACTCAAC.

We used the Nucmer software from the MUMmer 4 package (Marçais *et al.* 2018) to look for these telomere and centromere sequences in the scaffolds. For the purpose of counting distinct centromeric regions, we merged all copies of centromeres that were separated by less than 250** **Kbp. Overall, this process identified 33 centromeric regions, corresponding to the expected number of 33 chromosomes. During this process, we split scaffolds in 15 locations, based on telomere position and orientation and on not allowing any scaffold to contain more than one centromeric region. For example, the longest scaffold in the initial Dovetail scaffold set was ∼1.4** **Gbp in length and contained two copies of the centromere, at positions 760** **Mbp and 1.02** **Gbp (approximately). It contained telomeric sequences at 779 and 980** **Mbp, and we therefore split the scaffold at both of the telomere positions, which were presumably chromosome ends. The results of this splitting process, combined with screening for contaminants, which identified nine single-contig scaffolds totaling 46,650** **bp that contained human sequences, yielded an updated assembly, which we called version 2.2.

#### Repeat content annotation of assembly:

RepeatModeler v1.0.8 (Smit and Hubley 2008–2015) identified repeat families using RECON, RepeatScout, and TRF. The classified consensus library was further annotated with LTRclassifier (Monat *et al.* 2016). The resulting library was provided to RepeatMasker v.4.0.9 (Smit *et al.* 2019), to generate coordinates for the identified elements and to softmask the genome.

### Sequencing and assembling the transcriptome

#### RNA isolation and sequencing:

Total RNA was extracted from the following nine tissues collected from three individual coast redwoods: foliage from canopy sprouts, foliage from basal sprouts, sylleptic shoot tips, roots, 3-month-old seedling stems, megagametophytes, immature male cones, immature female cones, and mature male cones. Each tissue was immediately treated with liquid nitrogen upon collection, then stored in a −80°C freezer until extraction. All tissues were hand ground under liquid nitrogen with the addition of insoluble polyvinylpolypyrrolidone. The tissue was then lysed with the PureLink^®^ Plant RNA Reagent, clarified by centrifugation, isolated using two rounds of NaCl and chloroform wash, precipitated with isopropanol, washed with ethanol, and resuspended in nuclease-free water. All samples were purified using the Qiagen RNeasy^®^ MinElute^®^ Cleanup Kit and the RNase-free DNase Set.

The total RNA was first quantified with a Nanodrop 8000 (avg. concentration = 1596** **ng/μl, avg. A260/280** **=** **2.13; and avg. A260/230** **=** **2.06). Next, the concentration of pure RNA was determined using a Qubit 2.0 and the Qubit RNA BR assay kit (avg. concentration** **=** **1318** **ng/μl). Last, the RNA integrity was determined using the BIO RAD Experion^™^ automated electrophoresis station using the Experion^™^ RNA StdSens Analysis Kit (avg. RNA Quality Number** **=** **8.4). The RNA samples were sequenced on the Pacific Biosciences (PacBio) Isoform Sequencing (Iso-seq) platform carried out at the UC Davis Genome Center.

#### Assembly:

The Iso-seq data were processed as described by PacBio here: https://github.com/PacificBiosciences/IsoSeq/blob/master/isoseq-clustering.md. Briefly, the subreads were merged to generate one full-length circular consensus sequence (CCS). Primer artifacts were removed and the reads demultiplexed by library barcode. Polyadenylated tails and concatemers were trimmed and removed. Finally, the CCS reads were clustered into partial and full-length transcripts. Additional precautions were taken with IsoSeq Polish to generate consensus for each read cluster by generating per base quality values (QVs) for transcript consensus sequences.

### Integrating public transcriptomes

Currently, there are two RNA-seq libraries available, SRX7171900 (150** **bp PE) (Breidenbach *et al.* 2020), which represents a pooled needle tissue library from multiple clones, and ERX2099880 (100** **bp PE) (Matasci *et al.* 2014), which represents branch apex, including needle, tissue generated for the 1000 Plants (1KP) project. The Illumina sequencing reads were trimmed using Sickle v.1.33 (Joshi and Fass 2011) with the following parameters, quality threshold** **=** **30, length threshold** **=** **50. Trimmed reads were aligned to the coast redwood genome using HiSAT v.2.1.0 (Kim *et al.* 2019) with maximum intron length set to 2** **Mbp. Each aligned library was sorted by read name and subsequently merged using Samtools v.1.9 (Li *et al.* 2009). The merged aligned reads were used as one of the primary inputs for training the genome annotation pipeline, Braker2 v.2.0.5 (Hoff *et al.* 2016).

Three independent transcriptomes were concatenated and clustered at 98% identity with vsearch V.2.15.0 (Rognes *et al.* 2016). These included the Iso-seq transcriptome generated by us for our annotation, the short-read Illumina-derived transcriptome (Scott *et al.* 2016) available at https://datadryad.org/stash/dataset/doi:10.5061/dryad.7nb70, and the cold-stressed transcriptome (TSA: GIBU01000000) (Breidenbach *et al.* 2020). The combined resource was frame-selected based on ORF prediction from TransDecoder v.5.0.2. Transcripts with start codon, stop codon, or both (partials or complete) were selected and aligned to the genome with Gmap v. 2019 (Wu and Watanabe 2005) with the following parameters: minimum trimmed coverage and minimum identity** **=** **0.95 (–min-trimmed-coverage, –min-identity), full length proteins (−F), intron length** **=** **1,000,000 (−K), maximum total intron length** **=** **10,000,000 (−L), cds start** **=** **1 (−a), more sensitive search for canonical splicing (–cross-species), truncate alignment around full-length protein (−T), and return the single best alignment hit (−*n*** **=** **1). These CDS alignments were subject to constraints and quantification with gFACs (Caballero and Wegrzyn 2019). These constraints include: minimum exon and intron length** **=** **9** **bps, minimum cds length** **=** **100** **bps. The translated sequences of the transcriptomes generated previously were aligned to the coast redwood transcriptome using genome Threader v.1.6.6 (Gremme *et al.* 2005) with the following parameters: start codon, final stop codon, intron cut-out, dpminexonlen** **=** **20, dpminintronlen** **=** **9, gcmaxgapwidth** **=** **1,000,000, and gcmincoverage** **=** **80. These high-quality transcriptome and protein alignments served as an additional source of evidence when annotating the coast redwood genome.

### Structural annotation

#### Gene prediction with Braker:

The primary inputs provided to Braker included the soft-masked genome, RNA-seq alignments, and protein alignments. The merged RNA-seq alignment trains GeneMark-ET by using read pile up to estimate intron/exon boundaries of putative genes. The protein alignments are an optional component for structural annotations in Braker2. Although they do not directly impact the estimation of intron/exon boundaries during GeneMark-ET training, they contribute to the training of AUGUSTUS.

Given the large search space, high repeat content, and increased frequency of pseudogenes, it is a reasonable assumption that many of the predicted genes are false positives, fragmented, pseudogenes, or retroelements. Additional constraints are required to reduce the *ab initio* gene set. The *ab initio* genes must pass the following criteria through gFACs: complete open reading frame, a minimum exon and intron length of 9** **bps, and minimum CDS length of 100** **bps. Finally, the transcriptome sourced alignments are compared with the structurally filtered Braker models. Overlapping models are resolved through Bedtools v2.29 (Quinlan and Hall 2010).

In addition to structure, we leveraged functional information to determine a gene’s validity. We used EnTAP v0.10.4 (Hart *et al.* 2020) to identify genes that align to proteins from Uniprot (Apweiler *et al.* 2004), Plant RefSeq (Wheeler *et al.* 2005), and/or the proteomes of other gymnosperms. The custom gymnosperm database included proteins from: *P.* *abies, P. sitchensis, Ginkgo biloba, Cycas micholitzii, Gnetum montanum, Taxus baccata*, and *Abies sachalinensis*. Genes without sequence similarity search results, a single protein domain alignment, or a gene family assignment, are discarded. Removal of retroelements from the gene space was accomplished with InterPro Scan v.5.35-74.0 (Jones *et al.* 2014) using Pfam. This filter is coupled with the requirement that genes flagged as retroelements are at least 70% softmasked. Given that the mono-exonic gene space is highly inflated due to the presence of pseudogenes, the mono-exonic proteins are aligned to the multiexonic proteins requiring at least 75% identity and covering at least 70% of the mono-exonic gene.

### Orthogroup identification and enrichment for comparative genomics analysis

Proteomes for all available gymnosperms were downloaded from the forest genomics database TreeGenes (Falk *et al.* 2018). The *Amborella trichopoda* proteome (Chamala *et al.* 2013) was used as an outgroup to the gymnosperm taxa (accessed via Ensembl, Howe *et al.* 2020). Redundancy within a transcriptome was reduced by clustering the sequence at 80% identity with USEARCH (Edgar 2010). Species containing at least 15** **K unique sequences were evaluated for completeness with BUSCO v4.0.2 (Seppey *et al.* 2019) in protein mode using the Embryophyta lineage of OrthoDBv10 (Waterhouse *et al.* 2013). Species with at least 60% completeness were included in OrthoFinder (Emms and Kelly 2019) to identify orthogroups. Coast redwood genes in any gene family were clustered at 70% identity with USEARCH to determine their duplication status. Finally, coast redwood genes were aligned to the *Arabidopsis* proteome with Diamond v.0.9.36 (Hu and Kurgen 2019). As a result, this created a mapping between coast redwood genes and *Arabidopsis* genes which allowed for enrichment analysis through functional profiling available in Gprofiler (Raudvere *et al.* 2019). Families were examined for unique presence/absence as well as expansion/contraction determined from those with at least a 2-fold deviation for that family. Based on the unexpected ratio of Copia elements identified, genes with Copia insertions (within 3** **Kbp) were identified. A subset of these genes was enriched for flavonoid metabolic process and flavonoid biosynthesis and used as input for GeneMania (Montojo *et al.* 2014). Additional refinement of the network was done in Cytoscape (Su *et al.* 2014).

### Population genomics analyses

#### Nucleotide diversity and Fst estimates:

Ninety-two coast redwood samples from a greenhouse population that included accessions from the Kuser trial (Kuser *et al.* 1995), which cover the geographic and environmental natural range of the species were sequenced with 64,358 SNPs. Details on sequence capture, sequencing, SNP calling and filtering can be found in De La Torre *et al.* (2021). Fixation index (Fst) among populations was estimated using the method from Weir and Cockerham (1984) using the –weir-fst-pop option in VCFtools (Danecek *et al.* 2011). Data were analyzed using sliding windows with a step of 1000 (–fst-window-step option) and a window size of 10000 (–fst-window-size option) in VCFtools. Nucleotide diversity (PI) was also estimated using the sliding windows method used for Fst analyses (–window-pi and –window-pi-step options) in VCFtools. Nucleotide diversity and Fst estimates for coast redwood were compared with estimates from an unpublished study using similar targeted sequencing methods and number of SNPs in giant sequoia.

#### Estimating sequence divergence:

Nucleotide sequence divergence was estimated for the single-copy genes identified in the syntenic regions between coast redwood and giant sequoia and for the genes that were identified as unique to the coast redwood lineage (coast redwood, giant sequoia, and dawn redwood). The genes unique to the redwood lineage were found in multiple copies in coast redwood but in single copy in giant sequoia. Nucleotide sequences for 11 single-copy genes identified in the syntenic regions and 19 genes unique to the redwood lineage were aligned using MAFFT (Katoh and Standley 2014). For each alignment, Kimura 2-parameters distance was estimated using MEGA vX (Kumar *et al.* 2018) and pairwise synonymous (*dS*) and nonsynonymous (*dN*) substitution rates were calculated using PAML v.4.8 (Yang 2007). The parameters for the CODEML control file included runmode** **=** **−2 for pairwise comparisons, cleandata** **=** **0 for treating alignment gap as ambiguous characters, F3 × 4 model for determining codon frequencies along with default settings of initial values of 2 and 0.4 for estimating transition/transversion rate ratio and omega, respectively.

### Phylogenetic relationship of multicopy gene families

Phylogeny was inferred for the multicopy genes across the gene space to understand their relationships with other gymnosperms. The dataset included the genes that were present in multicopy (2 or 3 copies) in coast redwood but present as a single copy in other gymnosperms. With this criterion, 1700 genes were initially identified, which were further filtered based on their length (>300 amino acids) and shared at least 75% of the maximum sequence length. In addition, we removed gene variants resulting from possible tandem duplication from the analyses and that reduced the number of genes to 298. From these genes, we randomly sampled 150 and aligned their amino acid sequences using MUSCLE 3.8 (Edgar 2004) to infer phylogenetic relationships. For each alignment, maximum likelihood analysis was performed using IQTREE2 v.2.1.2 (Minh *et al.* 2020) with the substitution model selected based on Bayesian Information Criteria (BIC) using ModelFinder (Kalyaanamoorthy *et al.* 2017); branch support was assessed using nonparametric bootstrap from 300 pseudoreplicates.

## Results

### Sequencing and assembly

#### Sequencing data:

We used a hybrid sequencing and assembly strategy, combining accurate but relatively short Illumina paired-end reads with long, less-accurate reads from ONT sequencers. We used Dovetail’s Chicago and Hi-C library preparations to generate proximity ligation libraries that we used for scaffolding the assembled contigs. Illumina paired-end reads and ONT reads were used for building contigs, and reads from the Chicago and Hi-C libraries were only used for scaffolding. Sequencing data are summarized by library in Table 1.

#### Genome size estimation:

**Figure 1:**
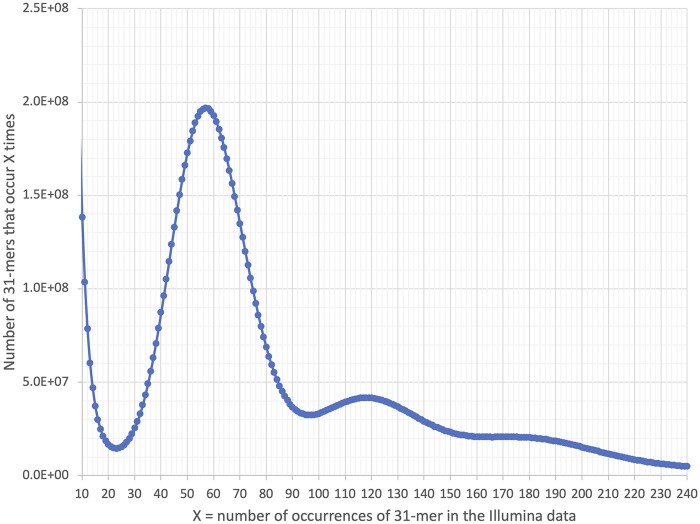
The distribution of 31-mers in coast redwood Illumina short-read data collected from a haploid sample. The primary peak is at *X* = 57. The number of 31-mers is estimated using the area under the curve, excluding the low-count k-mers that likely are due to errors in base-calling. The three peaks (X = 57: ∼118 and ∼180) reflect the hexaploid nature of the genome; these contain 31-mers that are identical in two or three of the subgenomes.

We estimated the haploid genome size by first counting 31-mers in the Illumina reads using the Jellyfish program (Marçais and Kingsford 2011). The main distribution peak in the 31-mer histogram (Figure 1) shows that the peak 31-mer coverage was 57. The number of 31-mers under the main peak is 1,503,160,425,741, which yields a genome size estimate of 1,503,160,425,741/57** **=** **∼26.5 Gbp. While this estimate is lower than the one based on flow cytometry, it should be close to the size of euchromatin (*i.e.*, noncentromeric DNA), as has been shown previously when using k-mers to estimate genome size (Vurture *et al.* 2017).

#### Hybrid assembly of super-reads and mega-reads:

The MaSuRCA assembler (Zimin *et al.* 2013) first extended all Illumina reads with unique extensions based on k-mers (*k*** **=** **127) present in the Illumina data, creating longer “super-reads.” The super-reads averaged 352** **bp (Table 2) and there were ∼213 million of them, compared to over 10 billion Illumina reads. Total CPU time for super-read construction was 11,472** **h, with maximum memory usage of 1.2** **TB. MaSuRCA then used the ONT reads as templates, aligning super-reads to them and producing paths of exactly overlapping super-reads that matched the underlying ONT read. These paths were then used to create mega-reads (Zimin *et al.* 2017), which are highly accurate replacements for the ONT reads. The mega-reads were slightly shorter than the ONT reads, because some ONT reads yielded two or more nonoverlapping mega-reads, as described previously. Note that because the ONT reads were produced from diploid DNA, about 50% of them came from a haplotype that was different from the haplotype represented by the Illumina reads. In some of these cases, insertion/deletion differences between the haplotypes may have resulted in alignment breaks and thus breaks in the mega-reads.

#### Assembly results:

The results from the initial assembly, the assembly after scaffolding with HiRise, and the final assembly (v2.2) of the coast redwood genome are shown in Table 3. The v1.0 assembly consisted predominantly of contigs with only minimal scaffolding, thus the N50 contig and scaffold numbers are very similar, 96,840** **bp for contigs and 109,446** **bp for scaffolds. The final assembly (v2.2) went through two rounds of HiRise scaffolding with Chicago and Hi-C data, followed by additional postprocessing. Scaffolding with Hi-C (proximity ligation) data increased the N50 scaffold size to 53** **Mbp. After examining the assembly for repeats, we noticed that several of the longest scaffolds in the v2.1 assembly contained centromeric and telomeric repeats in the middle of the scaffold (see *Materials and Methods*). We used these repeats to introduce 15 breaks in the scaffolds to obtain the final assembly (v2.2). This reduced the N50 scaffold size slightly, to ∼45** **Mbp. We also removed nine small contigs that we identified as contaminants. The v2.2 assembly has 33 scaffolds that contain centromeric regions, corresponding to the expected number of chromosomes. Names and sizes for these 33 scaffolds can be found in Supplementary Table S1. Four of the 33 also have telomeric repeats on one end, suggesting that they represent nearly complete chromosome arms. The 21 largest scaffolds in this set contain about 7** **Gbp of sequence.

**Table 3:** Assembly statistics for coast redwood, for the initial v1.0 assembly, the scaffolded v2.1 assembly, and the final v2.2 assembly

Assembly version	Total sequence (bp)	N50 contig size (bp)	N50 scaffold size (bp)	Number of contigs	Number of scaffolds
v1.0	26,454,318,454	96,840	109,446	548,924	517,860
v2.1	26,454,315,051	96,840	52,996,825	548,924	393,401
v2.2	26,454,268,401	96,840	44,944,384	548,915	393,407

For consistency, we computed the N50 sizes for contigs and scaffolds using 26.5 Gbp as the estimated genome size

During assembly of the Illumina data, we discovered reads representing two apparently novel fungal species, which were present in sufficient quantity to produce high-quality assemblies of each genome. The methods and results of assembling and identifying these species are described in Supplementary File S1.

### Transcriptome resources

The overall alignment rate of the RNA-seq needle libraries, SRX7171900 and ERX2099880, was 80% and 87.96%, respectively. The concatenated transcripts and fragments contained 409,920, 622,955, and 128,005 transcripts from the PacBio Iso-Seq long-read transcripts, de novo short read assembled needle transcripts from Breidenbach *et al.* (2020) and *de novo* short read assembled transcripts from Scott *et al.* (2016), respectively. After clustering, these transcripts collapsed into 131,842 PacBio Iso-Seq high quality transcripts, 568,778 transcripts from Breidenbach *et al.* (2020), and 97:764 from Scott *et al.* (2016). The combined reference transcriptome with 798,384 transcripts achieves 94.4% completeness when assessed with the BUSCO V5.0 Embryophyta lineage odb 10. After frame selection, 256,322 transcripts remained (90.8% BUSCO completeness) of which 202,414 were either full-length (132,195) or partial transcripts (70,219) (overall N50: 49,080** **bps, BUSCO completeness 90.6%). From the 202,414 transcripts 137:146 transcripts aligned to the genome at 95% identity and coverage. After constraining the alignment to be at least 100** **bps, setting minimum intron and exon sizes of 9** **bps, removing duplicates, and removing all incomplete protein-coding transcripts, 114,113 transcripts remained with 68.9% BUSCO completeness (Table 4).

**Table 4:** Transcriptome statistics: Illumina short-read and PacBio Iso-Seq needle tissue assemblies

Total transcripts	798,384
N50 (bp)	1,382
BUSCO completeness	94.4
Transcripts (frame selected)	256,322
Transcripts (filtered for completeness)	202,414
N50 (bp)	918
BUSCO completeness	90.8%
Total full-length	132,195
Aligned transcripts	114,113
N50 (bp)	933
BUSCO completeness	68.9%
Max intron length (bp)	1,977,916
Total multiexon transcripts	73,578
Total single-exon transcripts	40,535

### Gene annotation

#### Repeat discovery:

Soft masking of the genome resulted in approximately 18.7** **Gbp classified as repetitive (872 unique elements covering 70% of the genome). As is typical of conifers, this portion is dominated by transposable elements (TEs), which represent 59% (11.1** **Gb) of the repetitive elements, of which ∼80% (8.7** **Gb) are long terminal repeats (LTRs). Specifically, Gypsy and Copia elements are LTR-RT superfamilies that are prevalent across land plant phylogeny and constitute a significant portion of repetitive content (Casacuberta and Santiago 2003). Gypsy and Copia elements are differentially positioned in plant genomes (Ou and Jiang 2018). Copia elements are distributed proximal to genes, whereas Gypsy elements tend to be found in heterochromatic or pericentromeric regions (Baucom *et al.* 2009). In the coast redwood genome, these superfamilies span 8** **Gbp (2.06 and 5.94** **Gbp for Copia and Gypsy, respectively) and have a 2.88:1 ratio in favor of Copia. In coast redwood, 10,512 genes are associated with Copia-element insertions. These genes are primarily associated (enriched) for the phenylpropanoid and flavonoid biosynthesis pathways (Supplementary Figure S1). Among the gene ontology (GO) processes enriched, we note that response to biotic stress, bacterium, temperature, and heat were also dominant.

#### Protein coding gene identification:

The number of putative gene models retained for the final annotation was 118,906, of which 86,784 are multiexonic and 32,122 are mono-exonic. From these 118,906 gene models, 108,231 were constructed from aligned evidence, and 10,675 are derived exclusively from the transcriptome alignment. These transcripts extended 173,075 gene models from the initial 3.6 million gene models. Without the transcriptomic gene models, the BUSCO v.5.0 completeness score for the Embryophyta lineage was only 33% *vs* 67.8% with the addition of transcriptome alignment. Furthermore, characterization of the gene space using transcriptome alignment increased the overall average gene length by 93% (Table 5).

**Table 5:** Structural genome annotation summary

	Predicted gene space (Braker)	Filtered gene space (structure and function)	Final gene space (predictions and transcriptome)
Total genes	3,657,738	108,231	118,906
Average gene size (bp)	1,317	6,157	11,894
Average CDS length (bp)	663	1,151	1,150
Average number of exons	2.57	3.89	4.17
Average intron lengths (bp)	2,496	2,422	4,640
Maximum intron length (bp)	389,578	245,956	1,950,289
Total single-exon genes	3,048,838	31,110	32,122
Total multiexon genes	608,900	77,121	86,784
BUSCO completeness (%)	44.7	32.9	65.5

#### Functional characterization of genes:

Applying a stringent reciprocal functional annotation approach, we found 55,402 sequences had an alignment to the custom conifer database, NCBI’s Plant RefSeq, or UniProt database. The vast majority could be aligned to the precomputed EggNOG gene family database (117:714). The sequence alignments were primarily sourced from the conifer library with *Abies sachalinensis* representing the majority of the alignments followed by *Arabidopsis* and *Amborella* from the other databases. The GO terms applied from the gene family assignments and 96,006 (81%) could be assigned at least one term. The number of genes appearing to be novel is 591, when compared to existing public databases (Supplementary Table S1).

#### Exceptional introns:

To investigate functional patterns among genes with the largest introns, three length groups were analyzed. Coast redwood genes with introns ranging from 50 to 100** **Kbp were assessed for functional enrichment (1918 genes in total) (Supplementary Table S1). This set was enriched from a KEGG pathway perspective in both nicotinate and nicotinamide metabolism and fatty acid biosynthesis. Nicotinamide adenine dinucleotide (NAD) and its derivative NADP are well-conserved metabolites that orchestrate plant cellular redox homeostasis. These nucleotides also play vital roles in signaling via reactive oxygen species and in systems that respond to abiotic stressors, including: UV irradiation, salinity, heat shock, and drought (Manai *et al.* 2013). The independent GO enrichment identified the following processes: lipid metabolic processes, ncRNA processing, oxoacid metabolic processes, nitrogen, and organonitrogen compound metabolic processing, heterocyclic metabolic process, post translational gene silencing, and macromolecular methylation. In addition, terpenoid, amylopectin, and sulfur-compound biosynthesis were also enriched to a lesser degree. Among the 432 genes that contain introns ranging from 101 to 500** **Kbp, pathways supporting glucose sensing and signaling as well as flower development were enriched. The latter may relate more to seed development of other aspects of phenology of coast redwood. In terms of enriched GO biological processes, RNA processing was the predominant process observed outside of general metabolic processes. The number of genes with introns that exceed 500** **Kbp in length was 113. Among these, only one primary pathway and process were enriched and this relates to valyl-tRNA aminoacylation. Over-expression of genes from this pathway have been associated with increased resilience to abiotic stress in *Arabidopsis* (Richardson and Mullen 2011).

### Comparative genomics

#### Gene family analysis:

The gene family analysis, conducted with OrthoFinder, included 22 species and represented 841,087 proteins, of which 81% (680,419) could form putative gene families (orthogroups containing two or more proteins). The 35,647 resulting orthogroups represented 1155 species-specific orthogroups and 5665 with membership from all 22 species. Coast redwood genes could be found in about 44% of the total putative gene families. Only 54 orthogroups were identified as single-copy across the broad phylogenetic range assessed. Nearly 77% of the genes for coast redwood formed gene families, while 88% and 95% formed orthogroups for giant sequoia (from the genome annotation) and metasequoia (from a transcriptome), respectively (Supplementary Figure S2). There were 790 gene families identified as having three copies in coast redwood compared to a single copy in giant sequoia and metasequoia. Another 1075 families contained two copies in coast redwood compared to single copies in the same two species. Accounting for likely tandem gene duplications in coast redwood, there are 1394 gene families (288 of which contain tandem duplicates in coast redwood) that are single-copy across giant sequoia, metasequoia, and coast redwood. In addition, coast redwood had the largest number of species-specific gene families at 460. The resulting gene families were queried for patterns that may describe presence/absence and possible expansion/contraction in coast redwood, or among the Cupressaceae. Our summary of these results follows here and we provide a more detailed analysis in Supplementary File S2.

#### Coast redwood unique gene families:

We found 460 gene families present in coast redwood and absent in all other compared species. Gene families associated with abiotic and biotic stress were shown to form the majority of the unique families in coast redwood. A bifunctional abietadiene synthase (OG0022263) species-specific family was identified and is associated with defensive oleoresin formation in conifers in response to pest attack or physical injury (Celedon and Bohlmann 2019). These genes are specifically involved in diterpene olefins biosynthesis. In addition, a terpene synthase (OG0026422), involved in sesquiterpene, diterpene, and monoterpene biosynthesis, was present. An Ent-kaurene oxidase-like gene family (OG0035511), was identified and is involved in gibberellin biosynthesis. These diterpenoid hormones are precursors for most terpenoid products involved in defense. A flavonoid 3'-monooxygenase-like gene family (OG0026401) was noted. Flavonoid biosynthesis is an important branch of phenylalanine metabolism, and these compounds are regulated by the transcription profiles of several genes and manipulated by MYB transcription factors that are known to be expanded in conifers (Li 2014). Flavonoid components, such as flavone, flavonol, flavanols, procyanidins, and anthocyanins are modified in the presence of abiotic stress (in response to cell damage) (https://www.ncbi.nlm.nih.gov/pmc/articles/PMC4622623/). Among the broad category of genes known as lipases, a lipase class 3-like protein family (OG0035575) was identified. These were recently associated with activating salicylic acid-dependent defense responses in *Arabidopsis* (Lai *et al.* 2017).

#### Coast redwood gene families—absent in all other Cupressaceae:

We identified 1706 gene families present in coast redwood but not in other species of Cupressaceae. Related to plant stress and the proliferation of TEs common in conifers, Chromo (OG0011748; CHRromatin Organization MOdifier) as well as components of the FACT complex (OG0013343) and YT521 (OG0031005) were identified. These genes are associated with regulating chromatin structure and with epigenetic repression. A likely member of the WAX2 gene family (OG0013236), associated with the synthesis of the cuticle, was identified. Variations on WAX2 in plants, as studied in *Arabidopsis*, leads to variable structures and drought tolerance (less water loss) (Chen *et al.* 2003). Several xyloglucan endotransglycosylation families were identified (OG0015084; OG0020157; OG0025511; OG0026451; OG0034404; OG0035623; OG0012689). These genes cleave and regulate xyloglucan polymers, an essential constituent of the primary cell wall, and participate in cell-wall construction in growing tissues. In the case of mechanical stress, it may contribute to adaptive changes in morphogenesis by being recruited to alter the tissue’s tensil strength, or flexibility, enabling adaptation to mechanically stressful environments (Peña *et al.* 2004). Heparanase-like genes (OG0032932) are associated with abiotic stress in several plant species and also with structural remodeling (Ni *et al.* 2020). In terms of nutrient acquisition, a formamidase-like gene family was identified (OG0033463). It has been shown to induce proteoid roots in nutrient deprived soils (phosphorus and nitrogen) (Rath *et al.* 2010). In addition, a bark storage protein family (OG0032932), associated with nitrogen storage and differentially induced by nitrogen in *Populus* (Coleman *et al.* 1993), was noted.

#### Coast redwood expanded gene families:

We found that 7724 gene families expanded specifically in coast redwood. Metal-ion related genes were prevalent, including MATE transporters (OG0000181; OG0000326; OG0026382) and heavy metal associated proteins (OG0000170; OG0003233; OG0003549; OG0016719; OG0018282). Detoxification proteins and Transparent Testa-12 like proteins were also well represented. Detoxification 27(OG0012979; OG0000322), Detoxification 29 (OG0013597; OG0019025; OG0000383), Detoxification 40 (OG0003366), Detoxification 42 (OG0004540), and Detoxification 56 (OG0011608) were observed in large numbers. The related TESTA genes are regulators of flavonoid biosynthesis (Zhang and Shrader 2017). In addition, UDP-rhamnose-dependent rhamnosyltransferase (OG0000043), involved in anthocyanin modification, was observed (Hsu *et al.* 2017). The largest gene families in coast redwood were associated with disease resistance, and in specific, disease resistance protein RPM1-like (OG0000038). This protein specifically recognizes the AvrRpm1 type III effector avirulence protein from *Pseudomonas syringae* (Guttman and Greenberg 2001). Resistance proteins guard the plant against pathogens that contain an appropriate avirulence protein via an indirect interaction with this avirulence protein. Acidic endochitinase (OG0000527) is another very large family uniquely expanded in coast redwood. This protein functions as a defense against chitin containing fungal pathogens. Finally, oxophytodienoate reductase (OG0000500) was identified and is associated with pathogen response.

### Nucleotide diversity and Fst estimates

Results of the analyses of the exome capture data in 92 individuals suggest an average nucleotide diversity of 0.0001842 for coast redwood. This estimate is at least two orders of magnitude lower than the average nucleotide diversity per site for most outcrossing widespread gymnosperms (Savolainen and Pyhäjärvi 2007). When we compared coast redwood to its closest relative (giant sequoia), we found similar levels of average nucleotide diversity and average population Fst: 0.0001842 and 0.01127 for coast redwood, respectively, and 0.000089 and 0.01593 for giant sequoia, respectively.

### Origin of coast redwood hexaploidy

We aligned the entire coast redwood genome to the giant sequoia genome at the DNA level (see *Materials and* *Methods*), but the divergence is too great to detect genome-scale similarity. However, we were able to detect several large-scale regions of synteny, the largest of which spanned approximately 70 Mbp on giant sequoia chromosome 8 and coast redwood Scaffold_195192 (Supplementary Figure S3). The figure shows extensive rearrangements, but the overall synteny is clear.

Genes in this region that are still intact in both coast redwood and giant sequoia provided the basis for our analysis of the divergence between coast redwood and giant sequoia. By focusing on these genes, and independently on additional copies of the coast redwood genes, we hoped to determine whether the coast redwood genome duplications had occurred more or less recently than the divergence between coast redwood and giant sequoia.

An additional OrthoFinder analysis focused on the genes located in these syntenic regions: 416 genes from giant sequoia, 1197 genes from coast redwood, and available dawn redwood transcripts. This resulted in 11 single-copy gene families across the three species (Supplementary Table S2). The nucleotide variation across the 11 gene families assessed was 0.017–0.478 (1.7–48%). The low levels of variation among single-copy genes are expected. Those with greater variation could reflect differences in selection pressure or the inclusion of paralogs rather than orthologs in the analysis. Although the single-copy genes in the syntenic blocks are likely to be orthologs, it is conceivable that evolutionary processes such as gene duplication could result in paralogs. Similarly, *dS* and *dN* values are comparably low except for the four genes, OG0000185, OG0000195, OG0000251, and OG0000254, for which both *dS* and *dN* values have increased substantially. However, all have *dN/dS* values less than 1 indicating these genes may be under purifying selection.

Similarly, *dN*/*dS* < 1 was also observed for the majority of the genes unique to the redwood lineage, which includes multicopy sequences for coast redwood. The substitution rates remained consistently low for most genes, except for OG0018829, OG0018857, and OG0018879, for which increase in synonymous and nonsynonymous rates were noted for a variant sequence in the coast redwood. The overall nucleotide variation for these genes ranged from 0.024 to 0.56 with the majority of genes having <10% nucleotide variation between coast redwood and giant sequoia.

### Phylogenetic relationship of multicopy genes in coast redwood

**Figure 2:**
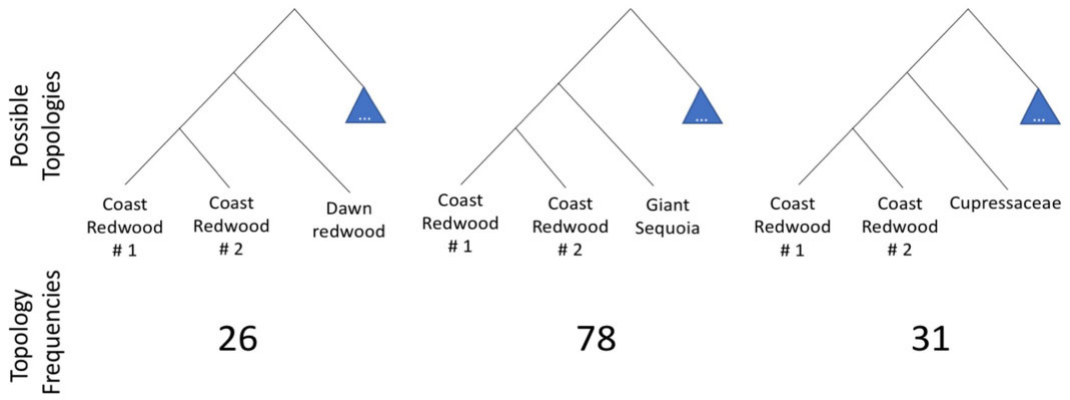
Frequency of different tree topologies when comparing multicopy genes in coast redwood to orthologous genes in giant sequoia, dawn redwood, and other species.

Phylogenetic analysis of multicopy genes, sourced from the full genome annotation of coast redwood and giant sequoia, resulted in maximum likelihood trees with coast redwood variants forming a clade for 135 genes, which includes 91 genes with two variants and 44 genes with three variants, out of 150 randomly selected genes (Supplementary Table S3). Among these 135 genes, 78 supported giant sequoia as sister to coast redwood, which was substantially higher compared to the 26 genes supporting dawn redwood as sister to coast redwood (Figure 2). The remaining 31 genes did not show clear relationships to either dawn redwood or giant sequoia. The large number of genes supporting giant sequoia as the closest relative to coast redwood was congruent with the previous finding based on the phylogenetic analysis using purely transcriptomic data (Scott *et al.* 2016). Furthermore, all trees showed the multiple coast redwood gene copies as closer to each other than to other species (Figure 2), which is in agreement with the autopolyploidy hypothesis previously postulated for the origin of coast redwood (Scott *et al.* 2016).

## Discussion

The large and repetitive polyploid coast redwood genome provided context for examining gene family dynamics when compared to other well-characterized plant genomes. Here, we noted substantial novelty in regard to both biotic and abiotic stress. Coast redwood is known for its pest and pathogen resistance and gene families associated with secondary metabolism and defense were enriched among the over 400 families that were not shared with the species compared. Among genes present specifically in coast redwood but absent only in other Cupressaceae, numerous genes associated with chromatin remodeling and methyltransferase activity were noted. The prevalence of these gene families, and their expression, is often associated with polyploids (Yuan *et al.* 2020). While polyploidy is common in species of genus *Juniperus*, also within the Cupressaceae, no members of this group were represented in this analysis. The enrichment signal may have been diluted with a more comprehensive species representation. Among families significantly expanded in coast redwood, defense-related genes were once again dominant, but we also specifically noted detoxification genes, TESTAs associated with flavonoid biosynthesis, and numerous MATE transporters. These genes have a range of functions, however, drought tolerance, metabolite transport, and development in response to light have been associated (Zhang and Shrader 2017; Volkov and Schwenke 2021).

Intron content and length regulate transcriptional efficiency and can be selected for in an evolutionary context (Heyn *et al.* 2015; Rose 2019). In addition, the position of the long intron relative to the start site is important (Parra *et al.* 2011). The maturation of a gene product may be delayed if long introns are present—this is known as intron delay (Heyn *et al.* 2015). Tissue-specific expression of genes with long introns has been noted in several species (Jeong *et al.* 2006; Baulin *et al.* 2020). The final protein-coding gene annotation of coast redwood contains genes with introns nearly 2** **Mbp in length which is consistent with observations made in other conifer genomes. The gene containing one of the longest introns is involved in regulation of defense response, regulation of jasmonic acid-mediated signaling pathway, and response to wounding. Among the categories of long introns examined, phenology, drought response, terpenoid production, and regulation of methylation were consistently observed as enriched when compared to the full annotated gene space.

As noted in all gymnosperm genomes, repeat content is high and LTRs dominate this landscape. In many plant genomes, Gypsy elements are traditionally associated with genome size evolution (and expansion) (Zhang *et al.* 2020). However, a recent study in *Arabidopsis arenosa*, an autotetraploid (with diploid individuals) noted that TE accumulation is higher in genic regions for the tetraploids, and specifically, Copias were enriched in and around genes associated with adaptive traits (and under positive, or relaxed purifying, selection) (Baduel *et al.* 2019). It has also been reported that *Ty1-copia* retrotransposons in plants are activated by both abiotic and biotic stressors (Joly-Lopez *et al.* 2017). These examples include both physical (UV irradiation and wounding) and chemical (salicylic acid) (Kimura *et al.* 2001). Among the over 10** **K genes associated with Copia-element insertions, phenylpropanoid and flavonoid biosynthesis pathways were noted as well as genes consistent with the enrichments observed in the gene family analysis.

Coast redwood, a mainly asexual species with occasional sexual reproduction showed very low levels of nucleotide diversity, based on exome capture data. This nucleotide diversity was at least two orders of magnitude lower than the average nucleotide diversity per site for most outcrossing widespread gymnosperms (Savolainen and Pyhäjärvi 2007) and similar to the mostly clonal giant duckweed *Spirodela polyrhiza*, which has some of the lowest values reported for plant species (Xu *et al.* 2019). Nucleotide diversity was also low for giant sequoia, suggesting the maintenance of all or most groves is required to ensure the long-term survival of the species.

Polyploidy is ubiquitous in angiosperms but is quite rare in gymnosperms (Khoshoo 1959; Ahuja 2005; Yang *et al.* 2012). The hexaploid nature of coast redwood was discovered independently by Japanese and American researchers in the 1940s (Hirayoshi and Nakamura 1943; Stebbins 1948). Stebbins (1948) proposed that the origin of hexaploid coast redwood was alloploidy (hybridization) based on cytogenetic analyses. Later, Ahuja and Neale (2002) modified the Stebbins hypothesis by suggesting that the origin was autoallopolyploidy or segmental polyploidy. The unresolved origin of coast redwood hexaploidy was not addressed again until Scott *et al.* (2016) proposed that this was just an autopolyploid event based on comparative genome sequencing in coast redwood, giant sequoia, and dawn redwood. We hoped that full genome sequencing in coast redwood and giant sequoia (Scott *et al.* 2020) might have resolved this issue once and for all. However, the analyses we performed (see *Results*) were not entirely conclusive but mostly support the autoplolyploid hypothesis of Scott *et al.* (2020). Ultimately, highly contiguous genome sequences in the three extant redwood species and even the possibility of sequencing extinct redwood species will resolve the origin and timing of the hexaploid nature of coast redwood.

## Data availability

The coast redwood v2.2 assembly was deposited at NCBI as GenBank accession VDFB02000000, BioProject PRJNA542879. The genome assembly, annotation, and functional assessment are also available in the TreeGenes database (https://treegenesdb.org/). The GSA Figshare portal (https://doi.org/10.25387/g3.16682680) was used to upload Supplementary Table S1: Comparative genomics workbook containing the functional annotation for all protein-coding genes, gene family analysis via OrthoFinder, gene family comparisons, enrichment analysis for long introns, and genes in proximity to copia LTRs; Supplementary Table S2: Single-copy gene family analysis workbook; and Supplementary Table S3: Multicopy gene family analysis workbook.
